# No relation between afferent facilitation induced by digital nerve stimulation and the latency of cutaneomuscular reflexes and somatosensory evoked magnetic fields

**DOI:** 10.3389/fnhum.2014.01023

**Published:** 2014-12-23

**Authors:** Sho Kojima, Hideaki Onishi, Kazuhiro Sugawara, Shota Miyaguchi, Hikari Kirimoto, Hiroyuki Tamaki, Hiroshi Shirozu, Shigeki Kameyama

**Affiliations:** ^1^Graduate School of Health and Welfare, Niigata University of Health and WelfareNiigata City, Niigata, Japan; ^2^Institute for Human Movement and Medical Sciences, Niigata University of Health and WelfareNiigata City, Niigata, Japan; ^3^Tokyo Bay Rehabilitation HospitalNarashino City, Chiba, Japan; ^4^Department of Neurosurgery, Nishi-Niigata Chuo National HospitalNiigata City, Niigata, Japan

**Keywords:** transcranial magnetic stimulation, digital nerve stimulation, afferent facilitation, cutaneomuscular reflex, somatosensory evoked magnetic fields

## Abstract

Primary motor cortex (M1) excitability can be assessed using transcranial magnetic stimulation (TMS) and can be modulated by a conditioning electrical stimulus delivered to a peripheral nerve prior to TMS. This is known as afferent facilitation (AF). The aim of this study was to determine whether AF can be induced by digital nerve stimulation and to evaluate the relation between the interstimulus interval (ISI) required for AF and the latency of the E2 component of the cutaneomuscular reflex (CMR) and the prominent somatosensory evoked field (SEF) deflection that occurs approximately 70 ms after digital nerve stimulation (P60m). Stimulation of the digital nerve of the right index finger was followed, at various time intervals, by single-pulse TMS applied to the contralateral hemisphere. The ISI between digital nerve stimulation and TMS was 20, 30, 40, 50, 60, 70, 80, 100, 140, 180, 200, or 220 ms. Single-pulse TMS was performed alone as a control. SEFs were recorded following digital nerve stimulation of the index finger, and the equivalent current dipole of prominent deflections that occurred around 70 ms after the stimulation was calculated. CMRs were recorded following digital nerve stimulation during muscle contraction. Motor evoked potentials (MEPs) were facilitated at an ISI between 50 and 100 ms in 11 of 13 subjects, and the facilitated MEP amplitude was larger than the unconditioned MEP amplitude (*p* < 0.01). There was no significant correlation between the ISI at which AF was maximal and the latency of the P60m component of the SEF (*r* = −0.50, *p* = 0.12) or the E2 component of the CMR (*r* = −0.54, *p* = 0.88). These results indicate that the precise ISI required for AF cannot be predicted using SEF or CMR.

## Introduction

Primary motor cortex (M1) excitability can be assessed using transcranial magnetic stimulation (TMS) and can be modulated by a conditioning electrical stimulus delivered to a peripheral nerve prior to TMS. The amplitude of motor evoked potentials (MEPs) in hand muscles elicited by TMS was decreased by conditioning electrical stimulation of the contralateral median nerve when the interstimulus interval (ISI) was 20–40 or 100–1000 ms (Chen et al., 1999; Tokimura et al., 2000; Sailer et al., 2003; Tamburin et al., 2005; Bikmullina et al., 2009). These inhibitory phenomena are known as short-interval afferent inhibition (SAI) and long-interval afferent inhibition (LAI), respectively. SAI and LAI are also induced by digital nerve stimulation (Chen et al., 1999; Tokimura et al., 2000; Sailer et al., 2003; Tamburin et al., 2005; Bikmullina et al., 2009). By contrast, M1 excitability is enhanced when the ISI is 45–80 ms, and this is known as afferent facilitation (AF). AF has been observed in MEPs recorded from hand muscles when median nerve stimulation was delivered 50–80 ms (Komori et al., 1992), 50–70 ms (Yokota et al., 1995), or 45–70 ms (Devanne et al., 2009) prior to TMS. Although AF was induced by electrical stimulation of the median nerve, it was not observed after electrical stimulation of the digital nerve (Komori et al., 1992; Devanne et al., 2009). However, Bikmullina et al. (2009) reported that AF was evoked by electrical stimulation of the digital nerve delivered 80 ms prior to TMS. It is therefore unclear whether cutaneous afferents stimulated by a conditioning electrical stimulus contribute to MEP facilitation under resting conditions.

Digital nerve stimulation during voluntary muscle contraction can induce successive periods of facilitation and inhibition of electromyographic (EMG) activity in the contracting muscle (Caccia et al., 1973; Jenner and Stephens, 1982; Maertens de Noordhout et al., 1992; Ridding and Rothwell, 1999). This cutaneous reflex can be measured stably and is known as the cutaneomuscular reflex (CMR). The CMR consists of an early period of facilitation (E1, 30–40 ms) followed by a period of inhibition (I1, 40–50 ms) and a later, more prominent facilitation (E2, 50–80 ms). The E2 component of the CMR is believed to involve a transcortical pathway, and lesion data support this hypothesis (Jenner and Stephens, 1982). During the early phase of the E2 component, while EMG activity is increased, MEPs elicited by TMS are suppressed (Maertens de Noordhout et al., 1992). However, when TMS is applied at the peak of the E2 component, the MEP amplitude is enhanced (Maertens de Noordhout et al., 1992). Although these studies suggest that digital nerve stimulation enhances M1 excitability, the relation between the latency of the peak of the E2 component of the CMR that is induced by digital nerve stimulation during muscle contraction and the ISI required to evoke AF that is induced by digital nerve stimulation under resting conditions is unclear.

Somatosensory evoked potentials (SEPs) or somatosensory evoked magnetic fields (SEFs) evoked by peripheral nerve stimulation have been widely used to investigate the physiology of normal somatosensory cortical processing. It is generally accepted that the peaks of prominent deflections occur at approximately 20 ms (N20), 26 ms (P30), 40 ms (N40), 42 ms (P45), and 67 ms (N75) after median nerve stimulation in SEPs (Nagamine et al., 1998) and 20 ms (N20m), 35 ms (P35m), and 60 ms (P60m) after median nerve stimulation in SEFs (Wikström et al., 1996; Nagamine et al., 1998; Hari and Forss, 1999; Huttunen et al., 2006, 2008; Huttunen and Lauronen, 2012). The peaks of prominent deflections in SEFs occur at approximately 25 ms (N20m), 41 ms (P35m), and 73 ms (P60m) after digital nerve stimulation (Onishi et al., 2013). In addition, it is widely accepted that the sources of N20m, P35m, and P60m are located in Brodmann area 3b, Brodmann area 3 or 4, and Brodmann area 1, 2 or 3, respectively (Forss et al., 1995; Wikström et al., 1996; Kimura et al., 1999; Inui et al., 2003; Huttunen et al., 2006). The variance of the latency for N20m and P35m across subjects was small, whereas that for P60m was relatively large (Wikström et al., 1996; Nagamine et al., 1998; Huttunen et al., 2006, 2008; Huttunen and Lauronen, 2012). In previous SAI studies, the ISI between peripheral nerve stimulation and TMS has been determined using the peak latencies of the SEP following peripheral nerve stimulation (Alle et al., 2009; Udupa et al., 2009; Fischer and Orth, 2011). These studies determined that the ISI required to induce SAI is the latency of N20 plus 2 or 3 ms (Alle et al., 2009; Udupa et al., 2009). Although there are some studies on AF (Komori et al., 1992; Yokota et al., 1995; Devanne et al., 2009), no studies have used the latency of SEPs or SEFs to determine the ISI between peripheral nerve stimulation and TMS. Therefore, we believe it is necessary to clarify the relation between the ISI required to induce AF and the latency of SEP or SEF peaks before AF is used in experiments.

We aimed to determine whether AF could be induced by digital nerve stimulation and to evaluate the relation between the ISI required for AF and the latency of the E2 component of the CMR and the P60m component of the SEF.

## Materials and methods

### Participants

Thirteen healthy, right-handed volunteers [age range, 22–29 years; mean ± standard deviation (SD) age, 23.5 ± 3.2 years] participated in this study. All subjects gave written informed consent. This study was approved by the ethics committee of Niigata University of Health and Welfare and conducted in accordance with the Declaration of Helsinki.

### EMG measurement

The subjects were seated comfortably in a chair. EMG was recorded from the right first dorsal interosseous (FDI) muscle using a silver/silver-chloride electrode in a belly-tendon montage. EMG signals were amplified (×100) by an amplifier (A-DL-720 •140; 4 Assist, Tokyo, Japan) and digitized at 2 kHz using an A/D converter (Power Lab 8/30; AD Instruments, Colorado Springs, CO, USA).

### Digital nerve stimulation

The right index finger was stimulated with ring electrodes at an intensity of three times the perceptual sensory threshold with a 0.2-ms square wave (SEN-8203; Nihon Kohden, Tokyo, Japan). The stimulating cathode electrode was placed immediately distal to the metacarpophalangeal joint and the anode electrode was placed immediately distal to the proximal interphalangeal joint (Chen et al., 1999; Tokimura et al., 2000).

### TMS

Monophasic-pulse TMS was delivered with a figure-of-eight-shaped coil (diameter, 95 mm) connected to a Magstim 200 (Magstim, Dyfed, UK). The coil was held with the handle pointing backward and laterally at approximately 45° to the sagittal plane. The optimal spot to elicit MEPs was carefully determined in each subject as the point where TMS consistently resulted in a large MEP in the right FDI, and the optimal coil position to evoke a stable MEP was marked on a cap worn by the subject (Miyaguchi et al., 2013). The TMS intensity used was the lowest stimulus intensity that induced an MEP with a peak-to-peak amplitude exceeding 1 mV in the relaxed FDI in at least 5 of 10 consecutive trials (Chen et al., 1999; Ridding and Rothwell, 1999).

### Afferent inhibition and AF measurement

**Figure 1:**
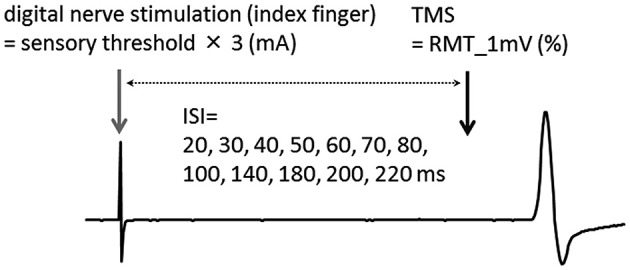
**Experimental protocol**. The interstimulus interval (ISI) between digital nerve stimulation and transcranial magnetic stimulation (TMS) was 20, 30, 40, 50, 60, 70, 80, 100, 140, 180, 200, or 220 ms. Single-pulse TMS was delivered alone (without a conditioning stimulus) as a control. RMT_1 mV; the lowest stimulus intensity that induced an motor evoked potential (MEP) with a peak-to-peak amplitude exceeding 1 mV.

Digital nerve stimulation of the index finger was followed, at various time intervals, by single-pulse TMS applied to the contralateral hemisphere. The ISI between digital nerve stimulation and TMS was 20, 30, 40, 50, 60, 70, 80, 100, 140, 180, 200, or 220 ms. Single-pulse TMS alone was used as a control (Figure 1). The TMS experiment consisted of 156 trials (12 trials at each ISI and 12 control trials) performed in a random order at a rate of 0.2 Hz.

### SEF recordings

Subjects were seated comfortably inside a magnetically shielded room (Tokin Ltd., Sendai, Japan) with their head firmly positioned inside a 306-channel whole-head magnetoencephalography (MEG) system (Vectorview; Elekta, Helsinki, Finland). This device consists of 204 planar-type, first-order gradiometers arranged as 102 pairs and 102 magnetometers. This configuration of gradiometers specifically detects the signal immediately above the source current. Continuous MEG signals were sampled at 1000 Hz using a band-pass filter between 0.03 and 330 Hz. To elicit SEFs, the right index finger was electrically stimulated using a pair of ring electrodes with a monophasic square-wave impulse of 0.2-ms duration at 0.5 Hz (Neuropack ∑ Nihon Kohden, Tokyo, Japan) in consideration of subject fatigue. The intensity of electrical stimulation was three times the perceptual sensory threshold. MEG data were obtained from 50 ms before to 200 ms after the stimulation, and a total of 200 epochs were averaged to identify the SEF. To analyze SEFs, the band-pass filter was set between 0.5 and 100 Hz and the 20-ms period of data preceding the stimulus was used as the baseline. Source Modeling software (Elekta, Helsinki, Finland) was used to estimate the time course of source activities. The sources of the components of interest in the SEFs were estimated as the equivalent current dipoles using a least-squares search using a subset of 16–18 channels over the area with the largest response.

### CMR

To record CMRs, the right index finger was electrically stimulated at three times the perceptual sensory threshold at 0.2 Hz during isometric contraction of the FDI muscle at 5% of the EMG maximum (Maertens de Noordhout et al., 1992; Ridding and Rothwell, 1999; Ridding et al., 2005). EMG data were rectified and averaged following 250 stimuli. Electrical stimulation is usually delivered at 3 Hz to record CMRs (Jenner and Stephens, 1982; Ridding and Rothwell, 1999; Ridding et al., 2005). However, we used 0.2 Hz to match the frequency of electrical stimulation used for afferent inhibition and AF measurements.

### Data analysis

MEP amplitudes were calculated from the peak-to-peak amplitude of the EMG and the maximum and minimum MEP amplitudes of the 12 trials in each condition were excluded. The SAI, LAI, and AF were calculated for each ISI (MEP ratio, conditioned MEP/unconditioned MEP). Statistical analysis was performed using PASW statistics software version 18 (SPSS; IBM, Armonk, NY, USA). Wilcoxon’s rank test was used to compare the amplitude of conditioned MEPs to the amplitude of unconditioned MEPs at each ISI. To further analyze MEP facilitation, we performed a within-subject analysis. In each subject, we identified the ISI between 50 and 100 ms that had the largest average conditioned MEP amplitude. We then compared the conditioned MEP amplitude at this ISI to the unconditioned MEP amplitude in this subject. The correlations between the ISI at which AF was maximal for each subject and the latency of the P60m component of the SEF and the E2 component of the CMR were assessed by Pearson’s correlation analysis. Differences were considered significant at *p* < 0.05 for all analyses. A summary of the protocol is shown in Table 1.

**Table 1:** **Summary of the study protocol**.

	AF	P60m	CMR
Stimulus location	Index finger	Index finger	Index finger
Stimulus frequency (Hz)	0.2	0.2	0.5
Resolution used in analysis (ms)	10	1	1

*AF, afferent facilitation; P60m, prominent somatosensory-evoked magnetic field deflection that occurs approximately 70 ms after digital nerve stimulation; CMR, cutaneomuscular reflex*.

## Results

**Figure 2:**
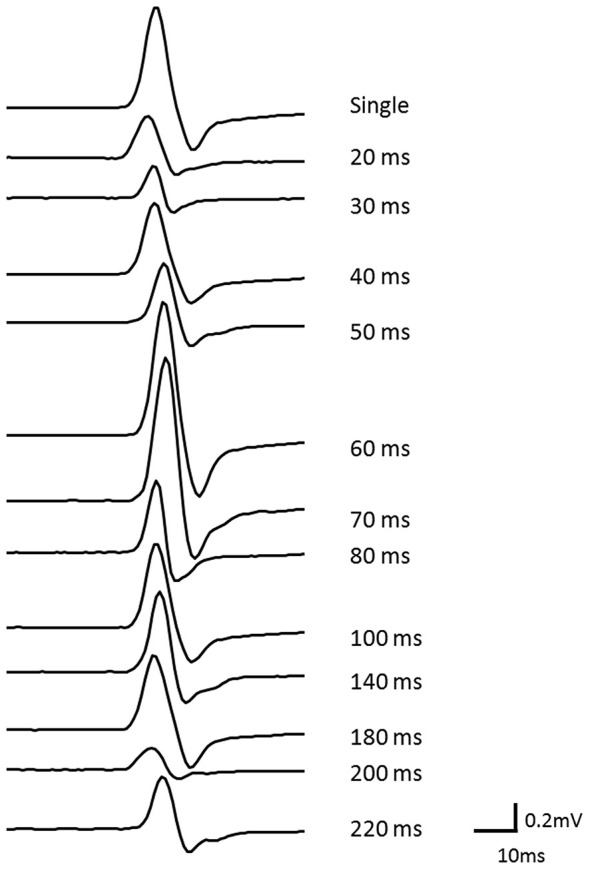
**The waveforms of conditioned MEPs elicited at each ISI for one subject**. The waveform of the MEP could be clearly observed at each ISI.

In afferent inhibition and AF measurements, the mean ± SD intensity of TMS was 49.7 ± 7.2% of the maximum stimulator output. Figure 2 shows the waveforms of the conditioned MEPs elicited at each ISI in one subject.

### Effects of ISI on MEP amplitude

**Figure 3:**
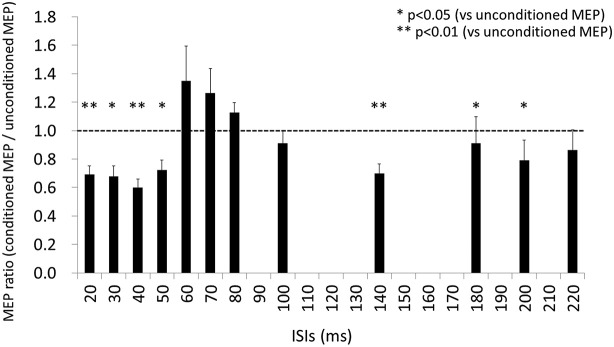
**Motor evoked potential (MEP) ratio for each ISI (ISI) between digital nerve stimulation and TMS**. At ISIs of 20, 30, 40, 50, 140, 180, and 200 ms, the amplitude of the conditioned MEP was significantly smaller than of the unconditioned MEP (*p* < 0.01 for 20, 40 and 140 ms; *p* < 0.05 for 30, 50, 180, and 200 ms). However, at the other ISIs (60, 70, 80, 100, and 220 ms) the amplitude of the conditioned MEP was similar to that of the unconditioned MEP.

**Figure 4:**
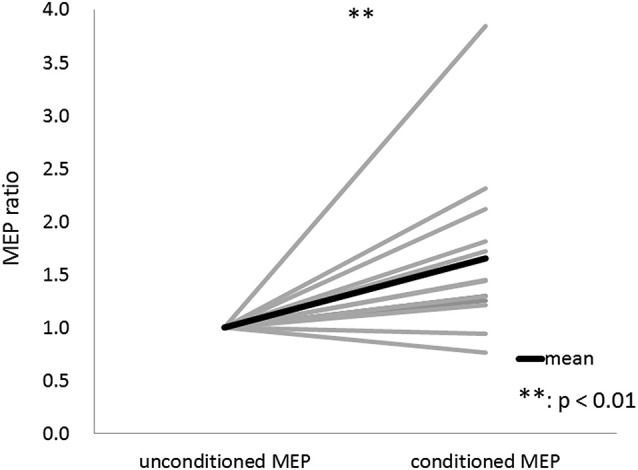
**Motor evoked potential (MEP) ratio for the ISI between 50 and 100 ms with the maximum conditioned MEP amplitude**. This ISI was determined separately for each subject. The conditioned MEP amplitude at this ISI was significantly larger than the unconditioned MEP amplitude in 11 of 13 subjects.

The results of Wilcoxon’s rank test illustrated that MEP amplitude was affected by the conditioning pulse for some but not all ISI. The mean ± standard error of the MEP ratio was 0.69 ± 0.06 at 20 ms, 0.68 ± 0.07 at 30 ms, 0.60 ± 0.06 at 40 ms, 0.72 ± 0.07 at 50 ms, 1.35 ± 0.24 at 60 ms, 1.26 ± 0.17 at 70 ms, 1.13 ± 0.07 at 80 ms, 0.91 ± 0.09 at 100 ms, 0.70 ± 0.07 at 140 ms, 0.91 ± 0.19 at 180 ms, 0.79 ± 0.14 at 200 ms, and 0.86 ± 0.14 at 220 ms (Figure 3). The amplitude of MEPs evoked by TMS delivered 20, 30, 40, 50, 140, 180, or 200 ms after digital nerve stimulation was significantly smaller than that of unconditioned MEPs (*p* < 0.01 for 20, 40 and 140 ms; *p* < 0.05 for 30, 50, 180, and 200 ms). However, the amplitude of MEPs evoked by TMS delivered 60, 70, 80, 100 or 220 ms after digital nerve stimulation was similar to that of unconditioned MEPs. MEP facilitation was confirmed for an ISI between 50 and 100 ms in 11 of 13 subjects and the conditioned MEP amplitude for this ISI was significantly larger than the unconditioned MEP amplitude (MEP ratio 1.65 ± 0.22; Figure 4). Table 2 shows the ISI between digital nerve stimulation and TMS at which MEP facilitation was greatest in each subject.

**Table 2:** **The age, height, interstimulus interval that evoked maximal AF, peak latency of P60m, and peak latency of the E2 component of the CMR for each subject**.

	Age (years)	Height (cm)	AF (ms)	P60m (ms)	CMR (ms)
Subject 1	23	180	60	66.3	72.6
Subject 2	22	161	70	72.7	65.7
Subject 3	29	169	60	58.6	72.3
Subject 4	22	167	70	70.7	69.5
Subject 5	22	161	80	61.5	57.9
Subject 6	22	161	60	78.6	71.1
Subject 7	22	160	–	69.1	77.8
Subject 8	24	173	70	80.3	67.3
Subject 9	23	168	60	70.7	79.6
Subject 10	22	151	60	69.0	73.8
Subject 11	22	165	60	83.0	71.1
Subject 12	26	172	–	82.0	67.6
Subject 13	27	172	100	57.0	68.7
Mean	23.5	166.2	68.2	70.7	70.4
SD	2.3	7.5	12.5	8.6	5.5

*AF, the interstimulus interval that evoked maximal afferent facilitation; P60m, the peak latency of the somatosensory-evoked magnetic field; CMR, cutaneomuscular reflex. ‘–’ indicates that afferent facilitation was not observed in this subject*.

### Relation between AF and SEF

**Figure 5:**
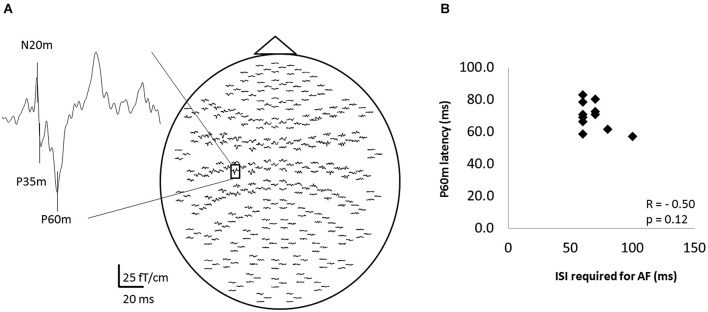
**The somatosensory evoked magnetic fields (SEFs) elicited from one subject, and the relation between the ISI that evoked maximal afferent facilitation (AF) and the latency of P60m in 11 of 13 subjects. (A)** The whole-scalp SEF waveforms evoked by digital nerve stimulation in one subject. SEF waveforms were clearly recorded in each subject. **(B)** The relation between the ISI that evoked maximal AF and the peak latency of the P60m component of the SEF. Each data point represents a different subject. There was no significant correlation between these variables.

Figure 5 shows the whole-scalp SEF waveforms evoked by digital nerve stimulation in one subject. The prominent deflections peaked at approximately 25 ms (N20m), 40 ms (P35m), and 70 ms (P60m) in the left primary sensorimotor cortex (Figure 5A). The mean ± SD latency of the P60m peak was 70.7 ± 8.6 ms (Table 2) and the latency was not significantly correlated with the ISI at which AF was maximal (*r* = −0.50, *p* = 0.12; Figure 5B).

### Relation between AF and CMR

**Figure 6:**
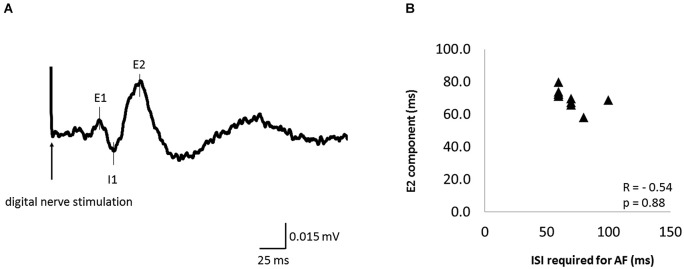
**The cutaneomuscular reflex (CMR) waveform elicited from one subject and the relation between the ISI that evoked maximal AF and CMR latency in 11 of 13 subjects. (A)** The CMR waveform evoked by digital nerve stimulation during muscle contraction in one subject. The waveform consisted of an early excitation (E1), an inhibition (I1), and a secondary excitation (E2). **(B)** The relation between the ISI that evoked maximal AF and the peak latency of the E2 component of the CMR. Each data point represents a different subject. There was no significant correlation between these variables.

Figure 6A shows the waveform of CMRs evoked by digital nerve stimulation during muscle contraction in one subject. This waveform consisted of early excitation (E1), inhibition (I1), and secondary excitation (E2). The mean ± SD latency of the peak of the E2 component was 70.4 ± 5.5 ms (Table 2) and the latency was not significantly correlated with the ISI at which AF was maximal (*r* = −0.54, *p* = 0.88; Figure 6B).

## Discussion

The main findings of the present study are that AF was induced by digital nerve stimulation, and that no significant correlations were observed between the ISI at which AF was maximal and the latency of the E2 component of the CMR or the prominent SEF deflection at approximately 70 ms after digital nerve stimulation.

AF was observed with an ISI of 50–100 ms in 11 of 13 subjects. At this ISI, the amplitude of the conditioned MEP was significantly larger than that of the unconditioned MEP. However, in group analyses, the MEP amplitude for each ISI within this range was not significantly different from the MEP amplitude of unconditioned stimuli. These results indicate that the ISI required to evoke MEP facilitation differed across subjects. In previous reports, MEP facilitation occurred when TMS was delivered 50–80 ms after median nerve stimulation (Devanne et al., 2009; Degardin et al., 2011; Mang et al., 2012), and AF was not observed after digital nerve stimulation (Komori et al., 1992; Devanne et al., 2009). Thus, Devanne et al. (2009) suggested that AF was induced by stimulation of a group Ia nerve or by activation of muscle spindles after muscle contraction. However, the intensity of digital nerve stimulation was not clear in their study. Bikmullina et al. (2009) reported that AF was induced by digital nerve stimulation with an intensity of three times the perceptual sensory threshold, but not by digital nerve stimulation with an intensity of one or two times the perceptual sensory threshold. AF was therefore modulated by the stimulus intensity, and it is possible that the intensity used by Devanne et al. (2009) was weaker than that required to evoke AF. Furthermore, Devanne et al. (2009) compared the average MEP amplitude calculated at fixed ISIs in all subjects. Therefore, it is possible that their study did not measure AF as effectively as we did in the present study. In our study, AF was evident in MEPs induced by digital nerve stimulation; this finding indicates that muscle contraction is not necessary for the induction of AF.

During the ISI required for AF induced by median nerve stimulation, H waves (Devanne et al., 2009) and MEPs induced by transcranial electrical stimulation were not facilitated (Classen et al., 2000). In addition, the ISI required for AF causes a decrease in short-interval intracortical inhibition and an increase in intracortical facilitation (Ridding and Rothwell, 1999; Devanne et al., 2009). Therefore, AF is believed to occur due to the modulation of intracortical excitability. Previous studies have reported that M1 is activated by digital nerve stimulation (Rosén and Asanuma, 1972; Lemon, 1979, 1981). Moreover, digital nerve stimulation during voluntary muscle contraction can induce CMR (Caccia et al., 1973; Jenner and Stephens, 1982; Maertens de Noordhout et al., 1992). The E2 component of the CMR, which peaks approximately 70 ms after digital nerve stimulation, is thought to be mediated at the cortical level (Jenner and Stephens, 1982). These studies demonstrate that digital nerve stimulation modulates M1 excitability. However, we observed no correlations between the peak latency of the E2 component of the CMR and the ISI that evoked maximal AF in this study. Therefore, it is considered that AF is induced by intracortical mechanisms different from those that induce the E2 component of the CMR during muscle contraction.

The equivalent current dipole of N20 or N20m following digital nerve stimulation has been estimated to occur in Brodmann area 3b of the primary sensory cortex (Forss et al., 1995; Kimura et al., 1999; Inui et al., 2003), and P60m reflects activation of Brodmann area 1 or 2 (Huttunen et al., 2006) or an inhibitory postsynaptic potential in Brodmann area 3 (Wikström et al., 1996). However, there is little direct information regarding the origin of P60m. Brodmann areas 1 and 2 have direct projections to Brodmann area 4 (Strick and Preston, 1978; Jones et al., 1979). If P60m reflects the activity of Brodmann areas 1 and 2, we then hypothesize that activation of these areas would either increase or decrease the excitability of Brodmann area 4. However, no significant correlation was observed between the latency of the P60m peak and the ISI that evoked maximal AF. These results indicate that the precise ISI required for AF is unpredictable before AF is used in an experiment.

In the present study, MEP amplitude decreased when TMS was delivered 20–50 or 140–200 ms after the digital nerve stimulus. These results are in agreement with previous studies, and these inhibitory phenomena have been called SAI and LAI, respectively (Chen et al., 1999; Classen et al., 2000; Tokimura et al., 2000; Kobayashi et al., 2003; Sailer et al., 2003). Our results suggest that SAI and LAI occur stably after electrical stimulation of either the digital nerve or a mixed nerve.

We investigated AF in 10-ms intervals to avoid fatiguing the subjects, but SEF and CMR latencies are defined with millisecond accuracy (Table 1). This difference in resolution is one of the limitations of this study. In future experiments we plan to probe AF in 1-ms intervals.

In conclusion, AF is induced by digital nerve stimulation and is evident when TMS is delivered between 50 ms and 100 ms after the digital nerve stimulation. However, the precise ISI required for AF is different in each subject, and there is no significant correlation between the ISI required for AF and the peak latency of the CMR or SEF after digital nerve stimulation. These results indicate that the precise ISI required for AF cannot be predicted using SEF or CMR.

## Conflict of interest statement

The authors declare that the research was conducted in the absence of any commercial or financial relationships that could be construed as a potential conflict of interest.
